# Breathable MOFs Layer on Atomically Grown 2D SnS_2_ for Stable and Selective Surface Activation

**DOI:** 10.1002/advs.202301002

**Published:** 2023-04-21

**Authors:** Gwang Su Kim, Yunsung Lim, Joonchul Shin, Jaegyun Yim, Sunghoon Hur, Hyun‐Cheol Song, Seung‐Hyub Baek, Seong Keun Kim, Jihan Kim, Chong‐Yun Kang, Ji‐Soo Jang

**Affiliations:** ^1^ Electronic Materials Research Center Korea Institute of Science and Technology (KIST) Seoul 02791 Republic of Korea; ^2^ KU‐KIST Graduate School of Converging Science and Technology Korea University 145 Anam‐ro Seongbuk‐gu Seoul 02841 Republic of Korea; ^3^ Department of Chemical and Biomolecular Engineering Korea Advanced Institute of Science and Technology (KAIST) 291, Daehak‐ro Yuseong‐gu Daejeon 34141 Republic of Korea; ^4^ KIST‐SKKU Carbon‐Neutral Research Center Sungkyunkwan University (SKKU) Suwon 16419 Republic of Korea; ^5^ Electronic Materials Research Center Korea Institute of Science and Technology (KIST) Seoul 02792 Republic of Korea; ^6^ School of Advanced Materials Science and Engineering Sungkyunkwan University (SKKU) Suwon 16419 Republic of Korea

**Keywords:** 2D materials, heterostructures, membranes, passivation

## Abstract

2D transition metal dichalcogenides (TMDs) have significant research interests in various novel applications due to their intriguing physicochemical properties. Notably, one of the 2D TMDs, SnS_2_, has superior chemiresistive sensing properties, including a planar crystal structure, a large surface‐to‐volume ratio, and a low electronic noise. However, the long‐term stability of SnS_2_ in humid conditions remains a critical shortcoming towards a significant degradation of sensitivity. Herein, it is demonstrated that the subsequent self‐assembly of zeolite imidazolate framework (ZIF‐8) can be achieved in situ growing on SnS_2_ nanoflakes as the homogeneous porous materials. ZIF‐8 layer on SnS_2_ allows the selective diffusion of target gas species, while effectively preventing the SnS_2_ from severe oxidative degradation. Molecular modeling such as molecular dynamic simulation and DFT calculation, further supports the mechanism of sensing stability and selectivity. From the results, the in situ grown ZIF‐8 porous membrane on 2D materials corroborates the generalizable strategy for durable and reliable high‐performance electronic applications of 2D materials.

## Graphical abstract

Here, ‘Sandwich‐like’ porous metal‐organic frameworks / 2D conductive heterostructures for realizing ultra‐stable and selective surface reactivity of conductive 2D materials as proven by chemical sensing case study and multiscale simulations are described for the first time.

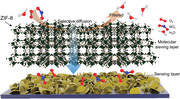

## 1 Introduction

2D materials (MoS_2_, SnS_2_, MXene, black phosphorus (BP), and graphene oxide) with intriguing physicochemical properties such as abundant reactive edge‐sites, tunable electrical properties, and mechanical flexibility, have been developed for broad applications in chemical sensing, electrocatalysis, filtration membrane, and energy storage system.^[^ ^1^ ^]^ Among various 2D materials, SnS_2_ (IV‐VI A group), which is a semiconducting layered structure with a large bandgap of ≈2.2 eV and has many advantages (low‐cost, nontoxic, and environmental friendly properties), can be catered well into the broad applications.^[^ ^2^ ^]^ Based on these interesting material properties of SnS_2_, 2D SnS_2_ is widely used as an active surface reactor with rapid, sensitive, and selective surface reactivity to various molecules including ions, gases, and biomolecules.^[^ ^3^ ^]^ For example, Song et al. developed a super‐selective NO_2_ reactor employing hierarchical SnS_2_ fabricated by ALD process,^[^ ^4^ ^]^ and Yang et al. developed biosensors based on SnS_2_ flakes for selective detection of glucose (2.5×10^−5^ to 1.1×10^−3^ m).^[^ ^5^ ^]^ Even though the outstanding and exceptional characteristics of SnS_2_ have been proved in many previous studies, its poor stability due to oxidation and degradation issues under ambient operation conditions limits the practical usage of SnS_2_.

To suppress the degradation of such 2D materials, various approaches such as encapsulation, protection, and molecular filtering techniques have been tried recently.^[^ ^3a^ ^]^ In particular, the development of “breathable” passivation layer with 2D materials has been suggested as the promising method to effectively activate the 2D surface reactivity and protect the harmful molecules (e.g., H_2_O and O_2_) that can facilitate oxidation/deterioration of 2D materials.^[^ ^6^ ^]^ In this point of view, nanoporous materials such as metal‐organic frameworks (MOFs), graphene oxide (GO), porous polymer, and zeolites can be incorporated with 2D materials so that the surface activity of 2D materials can be ensured. For example, sub‐10 nm holey GO was decorated on 2D MXene and BP to protect the harmful oxidants, while allowing the selective diffusion of target molecules.^[^ ^3a^ ^]^ However, non‐uniform pore distribution of GO‐based membrane (e.g., unexpected defects, and broad pore size distribution) has been regarded as a critical limitation for achieving reliable and reproducible “breathable” membranes. To overcome such limitations, we introduced thin MOFs layers having ordered porosity as an ideal breathable overlayer for SnS_2_ to preserve reliable surface activity with long‐term stability. It is noteworthy that the in situ formation of MOFs on heterogeneous substrates and pore size control are well established, which is advantageous for rationally designing the MOFs layers on desired 2D materials.^[^ ^6^, ^7^ ^]^

Furthermore, strong attachment of active materials on target device substrate is another critical hurdle for the practical use of electronic devices. For example, powder‐based nanomaterials synthesized by various synthetic methods (sacrificial templating route, electrospinning, and exfoliation method) have been typically drop‐coated on the desired substrate (e.g., interdigitated sensor substrate), thereby susceptible to fracture or delamination and being a big hurdle for the usage of nanomaterials in practical devices.^[^ ^8^ ^]^ To deal with these issues, the in situ growing methods with chemical reactions such as atomic layer deposition (ALD) and solution‐based self‐assembly were widely employed to ensure high durability due to strong bonding between active materials and heterogeneous target substrates.^[^ ^4^, ^9^ ^]^ Based on the previous achievements, we can expect that a rational design of sensing devices with improved performance and high mechanical stability could be achieved with the ALD or solution‐based self‐assembly methods.

In this work, we newly developed “Sandwich” like hybrid materials platform, 2D SnS_2_ layer covered by uniformly porous zeolite imidazolate framework (ZIF‐8), employing in situ growing methods. First of all, the hierarchical 2D SnS_2_ layer (1st layer) was directly grown on the Si‐based sensor substrate through ALD method, and as such, strong bonds between active material (SnS_2_ in this case) and the sensor substrate are able to be formed. Then, the in situ growing of ZIF‐8 was followed on the heterogeneous SnS_2_ layers, so the defect‐free ZIF‐8 covered the whole SnS_2_ layers. The top‐side of ZIF‐8 membrane and bottom‐side SnS_2_ play critical roles in molecular sieving and chemical detection, respectively. Due to the uniform pore distribution of ZIF‐8 layer on the SnS_2_ sensing layer, H_2_O molecules can be reliably blocked by ZIF‐8 breathable layer, while target NO_2_ molecules can selectively penetrate through the ZIF‐8 layer. Therefore, our hybrid materials exceptionally showed high stability and selective surface activity from the chemical sensing case study and the underlying mechanisms were further investigated at the atomistic level using theoretical calculations.

## 2 Results

### 2.1 Fabrication of ZIF‐8 Coated SnS_2_ Gas Sensor

**Figure 1:**
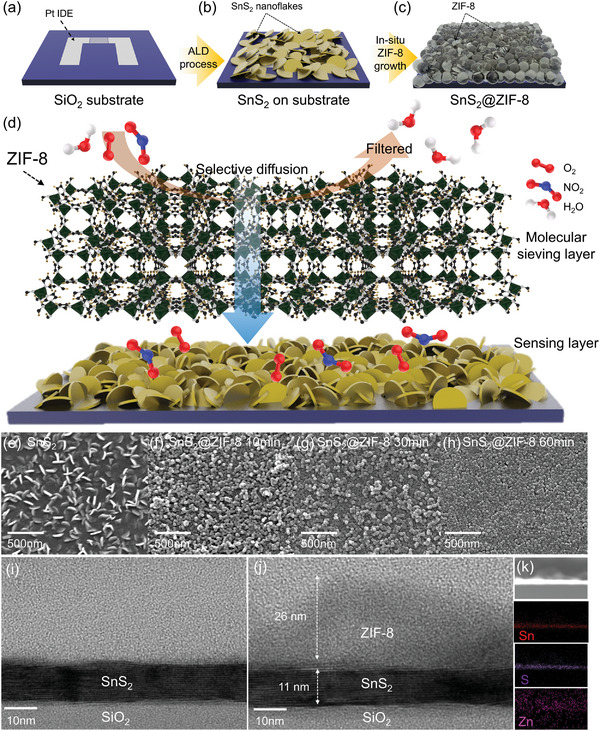
Conceptualization of ZIF‐8 coated SnS_2_ gas sensing platform with configuration analysis. a–c) Sequential synthesis protocols for SnS_2_@ZIF‐8. The SiO_2_ substrate was initially prepared and the SnS_2_ was constructed as nanoflakes morphology via PALD method. Then, the formation of ZIF‐8 layer was followed. d) Schematic illustration of mechanism and configuration of SnS_2_@ZIF‐8 in this work. SEM images of e) the pristine SnS_2_, and the SnS_2_@ZIF‐8 with different growth times of ZIF‐8 layer, f) 10, g) 30, and h) 60 min. Cross‐sectional view from TEM analysis for i) the pristine SnS_2_ and j) SnS_2_@ZIF‐8_10 min. k) EDS mapping analysis for the stacked layer.

As a standard protocol, we developed the ZIF‐8/SnS_2_ heterogeneous sensing platform by following protocols: i) atomically growth of SnS_2_ with hierarchical morphology on a sensor substrate, and ii) in situ growth of ZIF‐8 directly on the SnS_2_ layer (**Figure** **1**a–c). First, the plasma‐enhanced atomic layer deposition (PALD) method was conducted to directly deposit the SnS_2_ nanoflakes (SnS_2_ NFs) on Pt interdigitated electrode (IDE) (25 µm wide and 350 µm long lengths) as an active sensing layer (Figure 1a,b). As the deposition temperature and number of cycles are an important factor to control the sensor thickness and material phase,^[^ ^9^ ^]^ 240 °C and 200 cycles were respectively chosen for fabricating the hierarchical SnS_2_ NFs on sensing substrate, and detailed procedures for PALD are described in experimental section. Then, we selected ZIF‐8, which has ordered microspores of a suitable pore size (3.4 Å) for selective sieving of the desired gas molecules (e.g., kinetic diameter of NO_2_ = 0.33 nm), as a molecular sieving membrane (Figure 1c). Furthermore, the simple solution‐based synthetic process for ZIF‐8 is advantageous for the formation of defect‐free molecular sieving membrane on a desired substrate (e.g., SnS_2_ NFs). In a typical process, we immersed the SnS_2_ NFs on Pt‐IDE into a methanol‐based solution that contains the Zn precursor (Zn (NO_3_) 6H_2_O) and 2‐methylimidazole (mIM) linkers. Based on the heterogeneous nucleation and growth mechanism, the ZIF‐8 was uniformly grown on SnS_2_ layers with the controlled growth time of 10, 30, and 60 min, respectively referred to SnS_2_@ZIF‐8_10 min, SnS_2_@ZIF‐8_30 min, and SnS_2_@ZIF‐8_60 min. Therefore, as shown in Figure 1d, the final products are “sandwich” like SnS_2_@ZIF‐8 structures (SnS_2_ is sensing layer and ZIF‐8 is molecular sieving layer). To investigate the morphological behavior in respective samples, pristine SnS_2_, SnS_2_@ZIF‐8_10 min, SnS_2_@ZIF‐8_30 min, and SnS_2_@ZIF‐8_60 min were investigated by scanning electron microscopy (SEM). In the case of pristine SnS_2_, densely packed and hierarchical SnS_2_ NFs were grown on the sensor substrate, whereas in the SnS_2_@ZIF‐8 samples, the ZIF‐8 fully covered the SnS_2_ layers (Figure 1e–h). According to the previous reports, the different growth time of ZIF‐8 induces the different membrane thickness of ZIF‐8 on SnS_2_, that can affect the gas diffusion kinetics.^[^ ^10^ ^]^ Therefore, the optimum thickness of ZIF‐8 can be suggested through the surface reaction characteristics in chemical sensing measurement part. The morphological differences of prepared samples were further investigated by cross‐sectional transmission electron microscopy (TEM) analysis (Figure 1i,j). The samples for cross‐sectional TEM analysis for pristine SnS_2_ NFs and SnS_2_@ZIF‐8 were prepared by the focused ion beam milling method (details are in the experimental section). High‐resolution cross‐sectional TEM image of pristine SnS_2_ layer with 10–12 nm film thickness shows that SnS_2_ NFs are strongly attached to SiO_2_ substrate (Figure 1i). On the other hand, in the SnS_2_@ZIF‐8_10 min sample, the 26 nm ZIF‐8 layer on SnS_2_ was clearly observed (Figure 1j). The SEM images for the longer growth time of the ZIF‐8 layer (60 and 120 min) are shown in Figure S1, Supporting Information. The direct contact of ZIF‐8 (Zn component) with SnS_2_ film surface (Sn and S components) was further identified using Energy‐dispersive X‐ray spectroscopy (EDS) mapping analysis (Figure 1k).

### 2.2 Characterization of ZIF‐8 Coated SnS_2_

**Figure 2:**
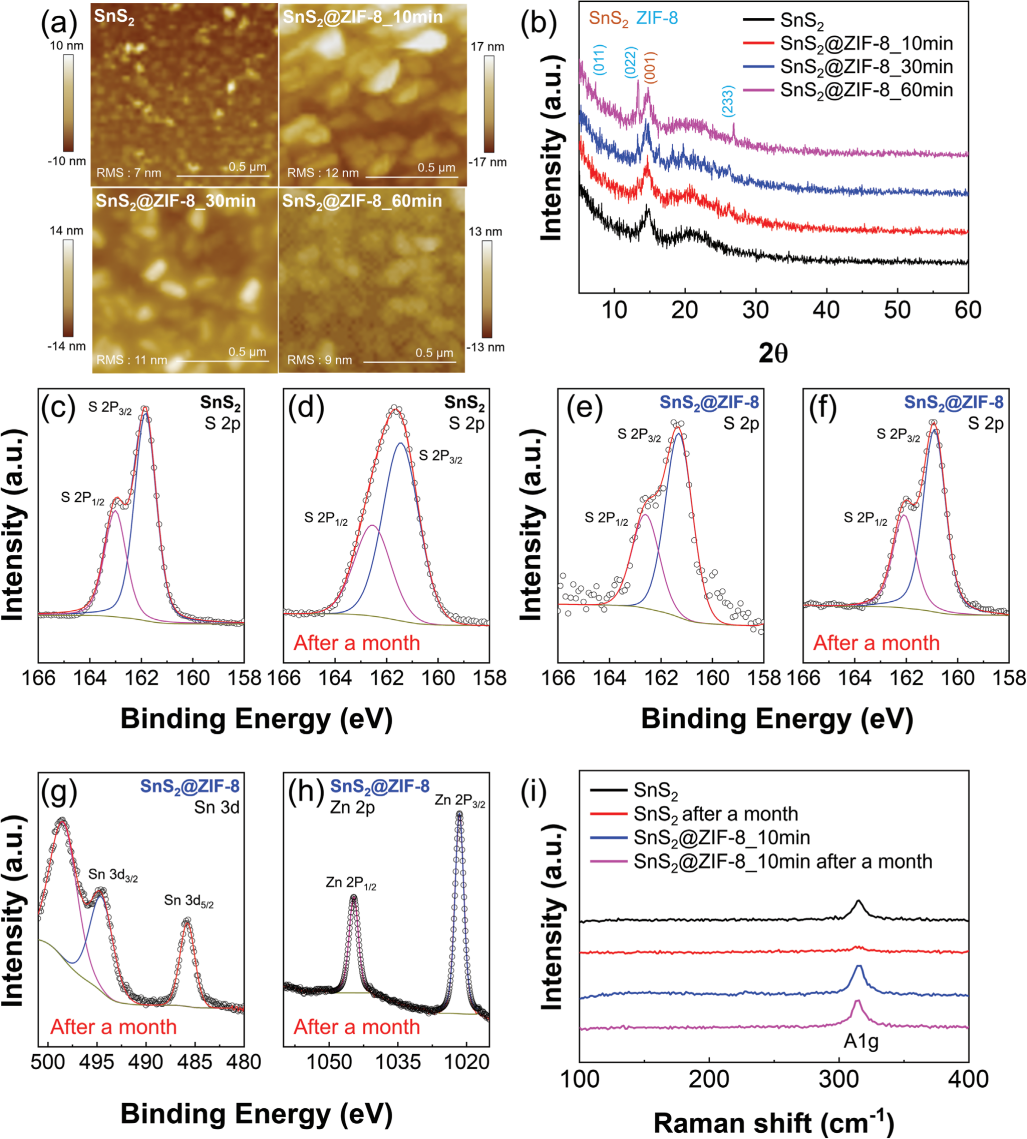
Surface characterization of the pristine SnS_2_ and SnS_2_@ZIF‐8. a) AFM images of the pristine SnS_2_ (upper left), SnS_2_@ZIF‐8_10 min (upper right), SnS_2_@ZIF‐8_30 min (lower left), and SnS_2_@ZIF‐8_60 min (lower right). Scale bar that presents nearby each image indicates RMS roughness values. b) XRD analysis of SnS_2_, SnS_2_@ZIF‐8_10 min, SnS_2_@ZIF‐8_30 min, and SnS_2_@ZIF‐8_60 min. XPS spectra of the pristine SnS_2_ and SnS_2_@ZIF‐8 in the vicinity of S 2p for the c,e) immediate analysis and d,f) analysis after a month. Additional XPS spectra of the SnS_2_@ZIF‐8 in the vicinity of g) Sn 3d and h) Zn 2p. i) Raman analysis for the pristine SnS_2_ and the SnS_2_@ZIF‐8_10 min with and without air exposure for a month.

The surface roughness analysis of the prepared samples was conducted with atomic force microscopy (AFM) images and root‐mean‐square (RMS) roughness values for a 1×1 µm topology maps (**Figure** **2**a). In the case of the pristine SnS_2_, RMS was analyzed to 7 nm due to the surface preferential growth of 2D SnS_2_ materials as observed in AFM (upper left image of Figure 2a). On the other hand, *σ*RMS of SnS_2_@ZIF‐8_10 min was 12 nm, and the RMS value gradually decreased as the ZIF‐8 growth time increased (11 nm for 30 min and 9 nm for 60 min cases). It means that the longer time ZIF‐8 growth time induces the close‐packed and smooth ZIF‐8 membrane on SnS_2_ NFs. In addition, the crystal structure of SnS_2_@ZIF‐8 was investigated by grazing‐incidence X‐ray diffraction (XRD) analysis (Figure 2b). Note that the following experiments were conducted with pristine SnS_2_ and SnS_2_@ZIF‐8 samples. We confirmed the successful growth of SnS_2_ by observing the main peak associated with the (001) plane of SnS_2_ at 15 degrees for all analyzed samples. For the SnS_2_@ZIF‐8_10 min and SnS_2_@ZIF‐8_30 min samples, ZIF‐8 related peaks were not clearly observed in XRD analysis due to the tiny amount of ZIF‐8 layer. On the other hand, the SnS_2_@ZIF‐8_60‐min sample has obvious diffraction peaks at 7.36°, 13.36°, and 26.88°, which are XRD patterns of ZIF‐8, confirming the successful synthesis of SnS_2_@ZIF‐8. Figure S2, Supporting Information, shows the FT‐IR spectrum analysis to confirm the existence of ZIF‐8 on the SnS_2_@ZIF‐8 samples. Pristine SnS_2_ did not show ZIF‐8 related peaks, while several bands were clearly observed for ZIF‐8 in the FT‐IR spectrum of SnS_2_@ZIF‐8 samples. The absorption band of 3132cm^−1^ is due to the C—H stretch of imidazole and the band at 1573cm^−1^ can be assigned to the C=N stretch mode. The absorption band in the ≈1100–1500cm^−1^ region is related to the C‐N stretch. We further conducted X‐ray photoelectron spectroscopy (XPS) analyses of the pristine SnS_2_ and SnS_2_@ZIF‐8_10 min to clearly investigate the ZIF‐8 membrane and its effect in the SnS_2_@ZIF‐8 (Figure 2c–h). In order to observe the surface stability corresponding to the existence of the ZIF‐8 layer, analyses were conducted for two different cases for the pristine SnS_2_ and SnS_2_@ZIF‐8, respectively: 1) samples immediately after fabrication, and 2) samples exposed to air for 1 month after fabrication. The irradiation XPS spectrum shows that the pristine SnS_2_ mainly consists of Sn, S, and C elements, and SnS_2_@ZIF‐8 contains additional Zn elements. For both pristine SnS_2_ and SnS_2_@ZIF‐8 samples, two peaks that contribute to Sn 3d_3/2_ and 3d_5/2_ (binding energy of 494.7 and 486.3 eV) were observed (Figure S3, Supporting Information). Similarly, the existence of both 2p_1/2_ and 2p_3/2_ show that the S 2p spin orbitals of the pristine SnS_2_ and SnS_2_@ZIF‐8 are split into two peaks at 162.4 and 161.2 eV. In addition, two peaks at 1021.7 and 1044.9 eV contribute to Zn 2p_3/2_ and 2p_1/2_ for SnS_2_@ZIF‐8. More importantly, as shown in Figure 2d,f, the full width at half maximum (FWHM) of S 2p_1/2_ and S 2p_3/2_ peaks increased for the pristine SnS_2_ sample after a month, while the FWHM of S 2p_1/2_ and S 2p_3/2_ peaks nearly preserved for the SnS_2_@ZIF‐8 sample even if it exposed to air. Considering that the FWHM values for the XPS can be calculated using the following equation: ΔE2 = ΔE2peak + ΔE2instrum (ΔE2peak: the natural line width of the peak, and ΔE2instrum: instrument resolution), the FWHM in XPS varies depending on the degree of oxidation for SnS_2_. Based on this reason, surface oxidation due to air exposure of the sample is considered to be the main reason for the increase in the FWHM of the pristine SnS_2_, and in the same vein, the surface oxidation can be effectively blocked for the SnS_2_@ZIF‐8. As a result, XPS data not only clearly confirmed that the successful deposition of the SnS_2_ sensing layers and ZIF‐8 molecular sieve membranes were achieved, but also indirectly imply that the ZIF‐8 sieve membranes can maintain the initial state of the SnS_2_ surface for a long duration (for a month in this case) by preventing surface oxidation. Furthermore, the crystal structures of the SnS_2_ and SnS_2_@ZIF‐8 were further investigated by Raman spectrum analysis (Figure 2i). In the Raman spectrum, the absorption peak at 314 cm^−1^ corresponds to the vibrational peak of A_1g_ of SnS_2_. For the samples analyzed as‐prepared SnS_2_ sample, a SnS_2_ A_1g_ peak was clearly observed in both SnS_2_ and SnS_2_@ZIF‐8 samples, but for the pristine SnS_2_, A_1g_ peak significantly decreased after a month while the peak remained for the SnS_2_@ZIF‐8 sample. It is noteworthy that the reduction of the Raman peak is mainly caused by surface oxidation, and as such, the Raman spectrum results also indirectly suggest that the porous ZIF‐8 sieving membrane prevents the surface oxidation of SnS_2_ which result is consistent with the results from the XPS spectrum analysis.

### 2.3 Surface Activity: Molecular Dynamics and Sensing Characteristics

**Figure 3:**
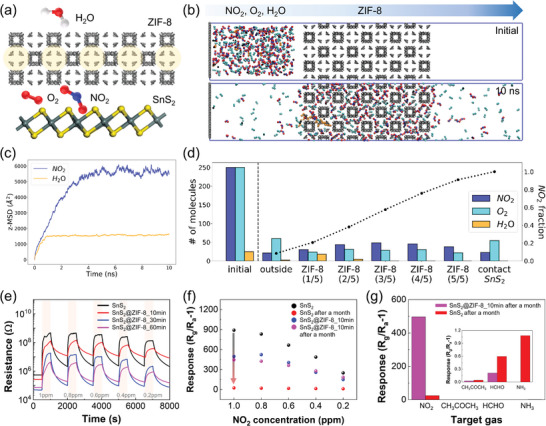
The role of the ZIF‐8 layer is to increase the stability and sensing performance of the SnS_2_ layer. a) Schematic illustration for the MD simulation. b) NO_2_, O_2_, and H_2_O gas molecules packed at the left part for the initial state (top) and they were diffused into the ZIF‐8 layer after 10 ns (bottom). Cyan color is O_2_ and orange color is H_2_O. c) z‐MSD for 50 NO_2_ (blue) and 50 H_2_O (orange) molecules. d) Quantifying the amounts of molecules that exist at the specific region (outside, ZIF‐8 layer, and contact to SnS_2_) in the overall system after 10 ns. Black line denotes cumulative NO_2_ fraction. e) Response transient for the pristine SnS_2_ (black), SnS_2_@ZIF‐8_10 min (red), SnS_2_@ZIF‐8_30 min (blue), and SnS_2_@ZIF‐8_60 min (magenta). f) Calculated response for the samples (pristine SnS_2_ and SnS_2_@ZIF‐8_10 min) with and without a month of air exposure in respect to the concentration of NO_2_ from 1 to 0.2 ppm. g) Compare the response of the pristine SnS_2_ (red) and the SnS_2_@ZIF‐8 sample (magenta) after a month for 4 different gas molecules (NO_2_, CH_3_COCH_3_, HCHO, and NH_3_).

Molecular dynamics (MD) simulations were conducted to observe the interaction between the ZIF‐8 layer and gas molecules at the molecular level, and to verify the increased stability and selective characteristics that are ascribed to the ZIF‐8 layer. Simulations were conducted with the gas mixture of three components (NO_2_, O_2_, and H_2_O) to determine whether the ZIF‐8 layer can act as a physical barrier to prevent the H_2_O molecules to reach the SnS_2_ surface. The simulations were run for a total of 10 ns at 300 K to ensure proper equilibration. Considering that NO_2_ and H_2_O molecules have high polarity, the linker rotations within the ZIF‐8 layer can be induced by the movement of these molecules, and as such, the gate‐opened ZIF‐8 structure (pore aperture enlarged from ca. 3.4 to ca. 4.0 Å) was considered in the simulations. As shown in **Figure** **3**a–d, among three gas molecule types that were initially packed on the left side of the ZIF‐8 layer, only the NO_2_ and O_2_ molecules penetrated through the layer and reached the SnS_2_ surface. This can be counterintuitive considering the fact that the H_2_O molecules possess the smallest kinetic diameter (NO_2_: 3.3 Å, O_2_: 3.46 Å, and H_2_O: 2.65 Å) among those three molecules. However, the hydrophobicity of the ZIF‐8 and the exceptionally high polarity of H_2_O molecules induce the clustering of the H_2_O molecules near the surface of the ZIF‐8 layer, and as such, these H_2_O clusters cannot infiltrate into the layer.^[^ ^11^ ^]^ Furthermore, to compare the mobility of NO_2_ and H_2_O molecules, additional MD simulations with the same number of NO_2_ and H_2_O molecules were conducted, and z‐directional mean squared displacements (z‐MSD) were calculated. Results from z‐MSD indicate saturation at ≈1600 Å^2^ for H_2_O while the z‐MSD of NO_2_ continuously increased up to ≈6000 Å^2^, which corroborates the reduced diffusivity of H_2_O observed in the previous MD simulations (Figure 3c and Figure S4, Supporting Information). Thus, from these results, it is confirmed that the water molecules cannot permeate through the ZIF‐8 layer. Since water is the most common mediator for electron transfer during the oxidation process, the oxidation of the SnS_2_ surface, which degrades the sensing performance, can be restrained by inhibiting the contact between the water molecules and the surface. Given that the thickness of the realistic ZIF‐8 layer would be much thicker than the modeled simulation, we can expect that the ZIF‐8 layer can be an effective physical protective layer that can maintain the performance of the SnS_2_ nanoflake.

To verify the phenomenon observed from the MD simulations, we conducted further experiments to identify sensing characteristics toward the target gas (NO_2_) and long‐term durability of SnS_2_@ZIF‐8 hybrid material. Initially, gases were exposed at room temperature and relative humidity of 40%, which is similar to the general atmospheric environment. The measurement process was controlled through a precise mass flow controller (MFC) system and was conducted based on a pressure of 1000 sccm. First, the detection characteristics between the pristine SnS_2_ and the SnS_2_@ZIF‐8 were investigated. In order to confirm the detection tendency according to the target gas concentration, the NO_2_ concentration was measured at intervals of 0.2 ppm from 1 to 0.2 ppm and room temperature. As shown in Figure 3e, one notable point is that the sensitivity gradually decreased as the ZIF‐8 growth time increased (Figures S5–S7, Supporting Information). However, even though the additional ZIF‐8 layer acts as a physical barrier for the gas penetration, the SnS_2_@ZIF‐8 still shows excellent sensing characteristics of 0.67 ppb limit of detection (LOD) even in the reduced sensitivity region (Figure S8, Supporting Information). Next, to confirm the long‐term stability of the sensor, samples in two different conditions were analyzed: 1) immediately after fabrication and 2) exposed to air for one month after fabrication. As demonstrated in the previous MD simulations, thin ZIF‐8 layer can be an effective physical barrier for humidity in atmosphere. In order to observe the ZIF‐8 effect, the NO_2_ detection characteristics for pristine SnS_2_ and SnS_2_@ZIF‐8 were compared (Figure 3f). Note that the response is defined as (*R*_g_/*R*_a_−1), where *R*_a_ and *R*_g_ denote the resistance of sensor in the absence and presence of the target gas. In the case of the pristine SnS_2_, it showed an excellent response of >891 for 1 ppm of NO_2_ for the initial state, while the response of pure SnS_2_ after a month dramatically decreased to 24 for 1 ppm of NO_2_. On the other hand, in the case of SnS_2_@ZIF‐8_10 min, the response for 1 ppm of NO_2_ was only slightly decreased from 497 to 447 even if the sample was exposed for a month (Figure 3f and Figure S9, Supporting Information). Furthermore, the sensing speed of SnS_2_@ZIF‐8_10 min was also preserved well (response time: 465 to 369 s and recovery time: 877 to 902 s), while pure SnS_2_ showed notable sensing speed variation (response time: 428 to 230 s and recovery time: 139 to 368 s) (Figure S10, Supporting Information). Then, we investigated the selective performance of pristine SnS_2_ versus the “sandwich‐like” SnS_2_@ZIF‐8 after a month to investigate their selective NO_2_ detection capability (Figure 3g and Figure S9, Supporting Information). As shown in Figure 3g, pristine SnS_2_ after a month showed relatively poor NO_2_ selectivity, while SnS_2_@ZIF‐8 after a month still showed outstanding NO_2_ detection capability with high selectivity. Further theoretical calculations and experiments were followed to support those results.

### 2.4 Selective Gas Detection: Theoretical Calculations and Sensing Characteristics

**Figure 4:**
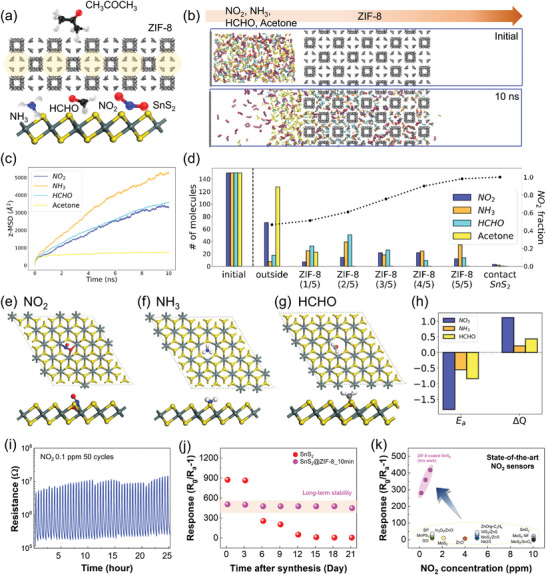
Selective sensing performance of SnS_2_@ZIF‐8 for NO_2_ gas. a) Schematic illustration for the MD simulation to filter out CH_3_COCH_3_ molecule. b) 4 gas molecules (NO_2_: blue/red, NH_3_: cyan, HCHO: orange, and CH_3_COCH_3_: yellow) packed at the left part for the initial state (top) and they moved in z‐direction toward the ZIF‐8 layer after 10 ns (bottom). c) z‐MSD for molecules. The color notation is the same as Figure 4b except for NO_2_ (blue). CH_3_COCH_3_ is referred to as Acetone for convenience. d) Quantifying the amounts of molecules that exist at the specific region of the system after 10 ns. Color notation is the same as Figure 4c and black line means cumulative NO_2_ fraction. e–g) Final configuration of (e) NO_2_, (f) NH_3_, and (g) HCHO at the SnS_2_ surface after geometry optimization. h) Adsorption energy and charge transfer between gas molecules and the SnS_2_ surface. The positive ΔQ means that the electrons are transported from the surface to the gas molecules. i) Long‐term stability of SnS_2_@ZIF‐8 toward cyclic exposure to 0.1 ppm NO_2_ gas. j) Time dependent NO_2_ 1 ppm sensing response test for SnS_2_ and SnS_2_@ZIF‐8. k) Comparison of the NO_2_ response of SnS_2_@ZIF‐8 with those of previously reported NO_2_ sensors.

To clearly demonstrate the selective NO_2_ detection characteristics of SnS_2_@ZIF‐8, additional MD simulations were conducted with the gas mixture of 4 components (NO_2_, NH_3_, HCHO, and CH_3_COCH_3_) to measure that the ZIF‐8 layer can work as a primary selective layer to filter out molecules with the large kinetic diameter such as CH_3_COCH_3_ (**Figure** **4**a). Similar to the previous MD simulations, the gas mixtures were initialized on the left part (outside) of the ZIF‐8 layer and the simulations were conducted for 10 ns at 300 K (Figure 4b). As CH_3_COCH_3_ has large kinetic diameters (4.5 Å), it cannot penetrate into the ZIF‐8 pore aperture, and as such, ca. 127 out of 150 molecules still remained outside of the layer after 10 ns. Even the remaining CH_3_COCH_3_ molecules did not pass through the 1/5 region of the ZIF‐8 layer. On the other hand, NO_2_ (3.3 Å), NH_3_ (3.46 Å), and HCHO (3.73 Å) that have relatively small kinetic diameters infiltrated into the ZIF‐8 filtering membrane and reached the SnS_2_ surface (Figure 4b–d). While the z‐directional mean squared displacements (z‐MSDs) of NO_2_, NH_3_, and HCHO molecules kept increasing for 10 ns, the motion of CH_3_COCH_3_ molecules with respect to z‐direction came to a standstill before 500 ps (Figure 4c). Therefore, we can expect that the ZIF‐8 layer can be an effective molecular sieve to filter CH_3_COCH_3_ and these results agree well with the experiments where there was nearly no response for CH_3_COCH_3_ at the SnS_2_ surface (Figure 3g and Figures S6 and S7, Supporting Information).

Next, DFT simulations were conducted to study the selective surface response of SnS_2_ with three filtered gas molecules from the previous MD simulations: NO_2_, NH_3_, and HCHO. Given that defects of the materials are inevitable during the experimental synthesis and can create a responsive site,^[^ ^12^ ^]^ the SnS_2_ monolayer with S‐vacancy was considered in this work to model the SnS_2_ nanoflake. Each gas molecule was located at the S‐vacancy site and adsorption energies were computed to measure binding strength between the SnS_2_ layer and gas molecules (Figure 4e–g). The uniquely large adsorption energy of NO_2_ (−1.83 eV) compared to NH_3_ (−0.56 eV) and HCHO (−0.84 eV) imply that chemisorption can be significant only between NO_2_ and SnS_2_ (Figure 4h). In addition, to verify and quantify the binding affinity from the electronic perspective, charge transfer between the gas molecules and the layer was computed using the Bader charge analysis. Although the electrons moved from surface to gas molecules in all three cases, the amounts of transferred electrons were quite different (Figure 4h). 1.12 e^−^ were transferred to NO_2_ molecules which is 5.33 times larger than NH_3_ (0.21 e^−^) and 2.60 times larger than HCHO (0.43 e^−^). The highest adsorption energy and electron migration between NO_2_ and the SnS_2_ surface imply that it is possible for the surface to preferentially detect NO_2_ among other small gas molecules, NH_3_ and HCHO. In the same vein, we can expect that the SnS_2_ surface will exhibit relatively weak reactivity in the order of HCHO and NH_3_, which matched with the experiment results. Therefore, these results from DFT calculations can strongly support the results from experiments that NO_2_ is the most responsive gas molecule with the SnS_2_ NFs at the atomistic level.

In addition to selective detection capability, the reliable sensing properties are also critical parameters for the practical use of our sensors. As shown in Figure 4i, SnS_2_@ZIF‐8 showed sufficient stability for analysis with only slight changes in base resistance and response even after repeated long‐term exposure to NO_2_ (repetitive 50 cycles). In addition, air‐exposed sensors were measured for NO_2_ 1 ppm every 3 days to confirm the long‐term stability of SnS_2_@ZIF‐8 compared to pristine SnS_2_ (Figure 4j and Figure S11, Supporting Information). As shown in Figure 4j, the NO_2_ detection characteristics of SnS_2_ significantly decreased by more than 90% from the 6 days, while SnS_2_@ZIF‐8 exhibited superior sensing stability even after 21 days. It should be noted that SnS_2_@ZIF‐8 exhibits superior NO_2_ sensing responses at room temperature comparable to the current state‐of‐the‐art 2D and/or semiconducting oxide NO_2_ sensors (Figure 4k and Table S1, Supporting Information).

## 3 Conclusion

In summary, we have successfully designed and developed ZIF‐8 having ordered porosity‐based breathable sieving membranes for ALD‐based 2D SnS_2_ materials, and demonstrated their effectiveness in chemical gas sensing applications. By intelligently using the high polarity of H_2_O molecules and hydrophobicity of the ZIF‐8 membrane, the exceptional H_2_O molecules clustering was formed at the interface of the ZIF‐8 layer, thus effectively blocking the water infiltration to 2D SnS_2_ sensing layer. Concurrently, targeting NO_2_, NH_3_, and HCHO gas molecules can simply diffuse into SnS_2_ sensing layer, however, the underlying SnS_2_ layer showed exceptional NO_2_ selectivity due to strong affinity between SnS_2_ and NO_2_ molecules. Such selectivity effect mechanism of SnS_2_@ZIF‐8 was clearly demonstrated by two‐types of simulation tools (e.g., DFT and MD simulation). In situ growing methods for 2D SnS_2_ and ZIF‐8 such as ALD and solution process induce the strong anchoring between SnS_2_@ZIF‐8 and sensor substrate, thus showing superior NO_2_ sensing stability. Moreover, the ALD‐driven hierarchical SnS_2_ layer showed superior NO_2_ sensing behavior compared to those of the state‐of‐the‐art RT NO_2_ sensing materials. We believe that our “Sandwich‐like” porous membrane/2D chemiresistive material heterostructures pave the new way for ultra‐stable and selective surface reactivity particularly optimized for sensors, catalysts, and electrochemical applications, where selective molecules transport is critical for material performances.

## 4 Experimental Section

### Device Fabrication

Pt/Ti (30 nm/30 nm thick)‐ IDEs were fabricated on a SiO_2_/Si substrate (1 µm/550 µm thick) with a 4‐inch wafer using a lift‐off procedure based on photolithography. The distances between each electrode were 5 µm and 20 electrodes were placed in a 1 mm × 1 mm area. The Pt‐IDEs patterned substrates were cleaned in acetone, ethanol, and deionized (DI) water, followed by drying under a flow of nitrogen gas.

### Synthesis of ZIF‐8 on SnS_2_ Nanoflakes

SnS_2_ NFs were deposited on the IDE by plasma‐enhanced ALD at 240 °C. As precursors for Sn and S, bis(1‐dimethylamino‐2‐methyl‐2‐propoxy)tin(ii) (Sn(dmamp)_2_, purity ≥ 99.9%) and H_2_S plasma were used. The H_2_S plasma was generated with an H_2_S (3.5%)/Ar flow of 400 sccm at a radio frequency power of 300 W. The ALD cycles were fabricated by supplying Sn(dmamp)_2_ for 2 s, purging for 10 s, supplying H_2_S plasma for 3 s, and purging for 20 s 100 times. ZIF‐8 was self‐assembled by dissolving 0.29 g of zinc nitrate hexahydrate and 0.6 g of 2‐methylimidazole in 35 mL of methanol. The prepared SnS_2_ was put into the solution at the same time as the powder was dissolved for 10, 30, 60, and 120 min to adjust the ZIF‐8 coating thickness. After synthesis, the ZIF‐8 coated SnS_2_ was washed once with methanol and dried at room temperature for 5 h.

### Molecular Dynamics

All of the MD simulations were conducted using the Large‐scale Atomic/Molecular Massively Parallel Simulator (LAMMPS) software.^[^ ^13^ ^]^ First, to simulate the function of the ZIF‐8 as a physical barrier for water vapor in the air, the large simulation box (3.4 nm × 3.4 nm × 18.6 nm) was prepared with a 2 × 2 × 4 supercell of ZIF‐8 structure. The gate‐opened ZIF‐8 structure was used in this work to facilitate the diffusion of gas species.^[^ ^14^ ^]^ ZIF‐8 structure was located at the midpoint of the simulation box and a gas mixture that consisted of 250 NO_2_, 250 O_2_, and 25 H_2_O molecules was placed at the left part of the ZIF‐8 layer. As periodic boundary conditions were applied for all three directions, the fixed wall composed of 1089 (33 × 33) pseudo‐atoms with only repulsive interactions was installed to induce diffusion of gas toward the ZIF‐8 layer and prevent not overflowing in the opposite z‐direction. Therefore, z = 0 to 5 nm that contains gas mixture at the initial time step would be the atmosphere, the ZIF‐8 layer exists as z = 5 to 13 nm, and z = 13 to 18.6 nm would be an interface of the ZIF‐8 and the SnS_2_ surface. To calculate z‐MSD, the gas mixture was adjusted for 50 NO_2_, 50 H_2_O, and 500 O_2_ molecules to preserve an equal contribution of target molecules (NO_2_ and H_2_O) to z‐MSD. All other conditions were conserved. Then, to simulate the molecular sieving effect of the ZIF‐8, only components of gas mixtures were changed and everything else was maintained. Gas mixtures were changed as 150 NO_2_, 150 NH_3_, 150 HCHO, and 150 CH_3_COCH_3_ molecules. For overall simulations, 5 different randomized gas mixture configurations were prepared and the final results have averaged the results from those 5 configurations for the reliability of the work. Every configuration was generated with the aid of moltemplate and packmol tools.^[^ ^15^ ^]^ The canonical NVT ensemble was endowed for the gas molecules with the Nose–Hoover thermostat to fix temperature as 300K.^[^ ^16^ ^]^ The simulation was conducted for 10 ns and z‐MSD was recorded for each gas type during overall simulations to quantify diffusivity of gas molecules. Universal Force Field (UFF) parameters were used to model the ZIF‐8 layer,^[^ ^17^ ^]^ and force field parameters reported from previous works were used for gas molecules (NO_2_ from Bourasseau et al.;^[^ ^18^ ^]^ O_2_ from Yu et al.;^[^ ^19^ ^]^ H_2_O from SPC/E model;^[^ ^20^ ^]^ NH_3_, and CH_3_COCH_3_ from TraPPE force field;^[^ ^21^ ^]^ HCHO from OPLS force field^[^ ^22^ ^]^). All molecules and the ZIF‐8 layer were regarded as rigid for convenience, and interatomic interactions were modeled as Lennard–Jones and Coulomb potential.

### Gas Sensing Measurements

The gas sensing properties of the pristine SnS_2_ NFs and SnS_2_@ZIF‐8 were examined in a quartz tube at room temperature. The gas flow was calibrated by mixing the dry air with water vapor using mass‐flow controller to be a constant flow rate of 1000 sccm. The sensor resistance was measured using a DC bias voltage of 0.5 V with a Keithley 2401 instrument and all data were recorded on a computer via the general‐purpose interface bus (GPIB) using LabVIEW software.

### DFT Calculations

To measure the sensing selectivity of the SnS_2_ nanoflake among infiltrated gas molecules (NO_2_, NH_3_, and HCHO), DFT calculations were conducted. All of the DFT calculations were conducted using the Vienna Ab initio Simulation Program (VASP) software v5.4.1.^[^ ^23^ ^]^ As with the previous work,^[^ ^24^ ^]^ the defective monolayer SnS_2_ was modeled by eliminating a single sulfur atom from 5 × 5 × 1 supercell SnS_2_ (25 Sn atoms and 49 S atoms). Considering that the periodic boundary conditions in all directions were considered, the sulfur located in the midpoint of the SnS_2_ was eliminated to minimize the interaction between the S‐vacancy sites. In the same vein, the vacuum layer with a thickness of 20 Å was introduced above the SnS_2_ to restrict any self‐interaction. The projector augmented wave (PAW) potentials were used to consider the interactions between the ions and electrons,^[^ ^25^ ^]^ and the generalized gradient approximation (GGA) functional of Perdew, Burke, and Ernzerhof (PBE) was used to calculate the exchange‐correlation potentials.^[^ ^26^ ^]^ The kinetic energy cut‐off was set as 400 eV. Conjugate‐gradient algorithm was used for the geometry optimizations until the forces became less than 0.02 eV Å^−1^ and the criterion for electronic self‐consistent loop was set as 1 × 10^−5^ eV. During the calculations, the Brillouin zone was sampled as the 5 × 5 × 1 Γ‐centered k‐points grid. Considering that the binding between the SnS_2_ surface and the gas molecules would occur in the S‐vacancy site, the gas molecules were put just above the S‐vacancy site as the initial configurations for the geometry optimization. Then, the adsorption energy (*E*_a_) between the SnS_2_ slab and gas molecules was computed via the following equations: *E*_a_ = *E*_slab+gas_ − (*E*_slab_ + *E*_gas_). In addition, Bader charge analysis was conducted to quantify charge transfer.^[^ ^27^ ^]^

### Characterization

The morphology of the fabricated SnS_2_ thin films was observed using a field‐emission scanning electron microscope (inspect F50) with an acceleration voltage of 15 kV and a working distance of 10 nm. Glancing angle XRD (D8 advance) and Raman spectroscopy (inVia Raman Microscope, Reinshaw) were utilized to verify the phase of the grown films. The XRD measurements were carried out at a scan rate of 4° min^−1^ at 10°–50°, with CuK*α* radiation (1.5418 Å wavelength) as the X‐ray source at a fixed incident angle of 2°. The chemical binding states of the SnS_2_ thin films were examined by X‐ray photoelectron spectroscopy (XPS, 5000 VersaProbe). The binding energies were calibrated against the Sn 3d peak (486 eV) and S 2p (161.8 and 162.9 eV) using a monochromated AlK*α* X‐ray source (1486.6 eV). The surface morphology and roughness of the deposited SnS_2_ and ZIF‐8 coated SnS_2_ were determined by non‐contact mode AFM (XE‐100). FT‐IR analysis was used to identify the composition and functional group strength of SnS_2_ and SnS_2_@ZIF‐8. The FT‐IR spectrum was analyzed using ATR (Attenuated Total Reflectance) Technique of Perkin Elmer FT‐IR spectrometer from 400 to 4000cm^−1^ in an average of 64 scans at 4cm^−1^ resolution.

## Conflict of Interest

The authors declare no conflict of interest.

## Supporting information

Supporting Information

## Data Availability

The data that support the findings of this study are available from the corresponding author upon reasonable request.
