# Supplementary material for: Breathable MOFs Layer on Atomically Grown 2D SnS2 for Stable and Selective Surface Activation
Source: Adv Sci (Weinh). 2023 Apr 21;10(17):2301002. doi: 10.1002/advs.202301002 (PMC10265055; doi:10.1002/advs.202301002)
Supplement: Supplementary file 1 — Supporting Information [file ADVS-10-2301002-s001.pdf]

ADVANCED SCIENCE
Open Access

# Supporting Information

for *Adv. Sci.*, DOI 10.1002/advs.202301002

Breathable MOFs Layer on Atomically Grown 2D $SnS_2$ for Stable and Selective Surface Activation

*Gwang Su Kim, Yunsung Lim, Joonchul Shin, Jaegyun Yim, Sunghoon Hur, Hyun-Cheol Song, Seung-Hyub Baek, Seong Keun Kim, Jihan Kim*, Chong-Yun Kang* and Ji-Soo Jang**

# Supporting Information

**Breathable MOFs layer on atomically grown 2D SnS2 for stable and selective surface activation**


**Authors**

Gwang Su Kim¶, Yunsung Lim¶, Joonchul Shin, Jaegyun Yim, Sunghoon Hur, Hyun-Cheol Song, Seung-Hyub Baek, Seong Keun Kim, Jihan Kim*, Chong-Yun Kang*, Ji-Soo Jang*

**Affiliations**

G.S. Kim, J.-S. Jang, J.C. Shin, J. Yim, Prof. C.-Y. Kang

Electronic Materials Research Center Korea Institute of Science and Technology (KIST)

Seoul 02791, Republic of Korea

G.S. Kim, J. Yim, S. K. Kim, C.-Y. Kang

KU-KIST Graduate School of Converging Science and Technology Korea University

145 Anam-ro, Seongbuk-gu, Seoul 02841, Republic of Korea

Y.Lim, Prof. J. Kim

Department of Chemical and Biomolecular Engineering, Korea Advanced Institute of Science and Technology (KAIST), 291, Daehak-ro, Yuseong-gu, Daejeon 34141, Republic of Korea

*E-mail: jihankim@kaist.ac.kr, cykang@kist.re.kr, wkdwltn92@kist.re.kr

¶These authors contributed equally:


**Keywords**: 2D, heterostructure, membrane, passivation

# Table of contents

**Figure S1.** SEM images for $SnS_2$@ZIF-8 with ZIF-8 growth time of 60 min and 120 min.

**Figure S2.** FT-IR spectra of $SiO_2$ substrate, pristine $SnS_2$, $SnS_2$@ZIF-8 10min, $SnS_2$@ZIF-8 30min, and $SnS_2$@ZIF-8 60min.

**Figure S3.** XPS analysis for the pristine $SnS_2$ and the $SnS_2$@ZIF-8 in the initial state and the after a month of air exposure.

**Figure S4.** Additional MD simulations to calculate z-MSD of $NO_2$ and $H_2O$ molecules.

**Figure S5.** Calculated response for the samples in respect to the concentration of $NO_2$ from 1 to 0.2 ppm.

**Figure S6**. Dynamic response of the pristine $SnS_2$ and $SnS_2$@ZIF-8 with various growth time toward 4 different gas molecules ($CH_3COCH_3$, $NO_2$, HCHO, and $NH_3$).

**Figure S7.** Changes in response according to the growth time difference for the ZIF-8 layer.

**Figure S8.** Response versus $NO_2$ concentration for the $SnS_2$@ZIF-8_10min sample after a month of air exposure.

**Figure S9**. Changes in response according to the prolonged air exposure.

**Figure S10**. Response and recovery time data for the pristine and ZIF-8 modified sample.

**Figure S11.** Time dependent $NO_2$ sensing response test.

**Table S1.** Comparison of previously reported $NO_2$ gas sensor.

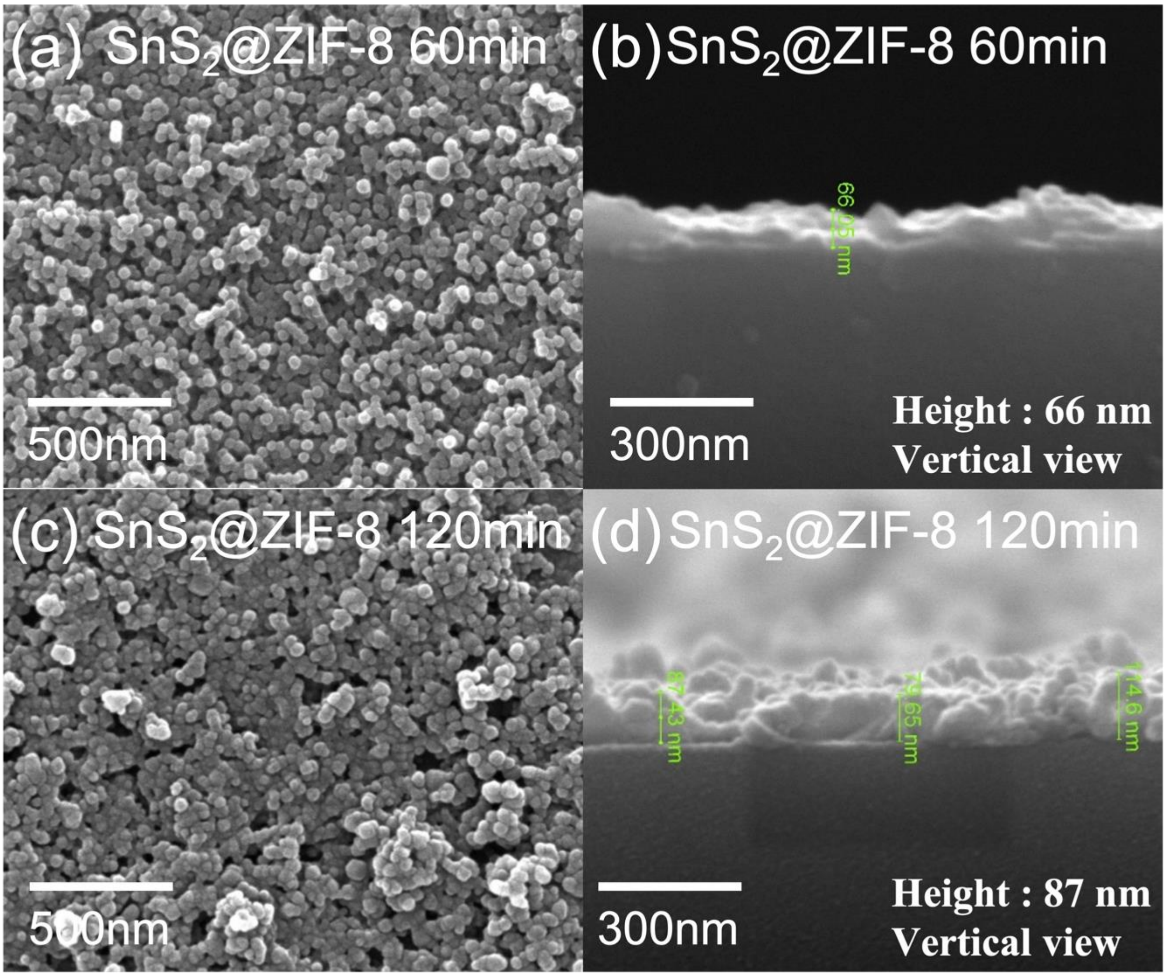


**Figure S1.** SEM images for $SnS_2$@ZIF-8 with ZIF-8 growth time of 60 min and 120 min. (a) SEM image and (b) cross-sectional SEM image for $SnS_2$@ZIF-8_60 min sample. (c) SEM image and (b) cross-sectional SEM for $SnS_2$@ZIF-8_120 min sample.

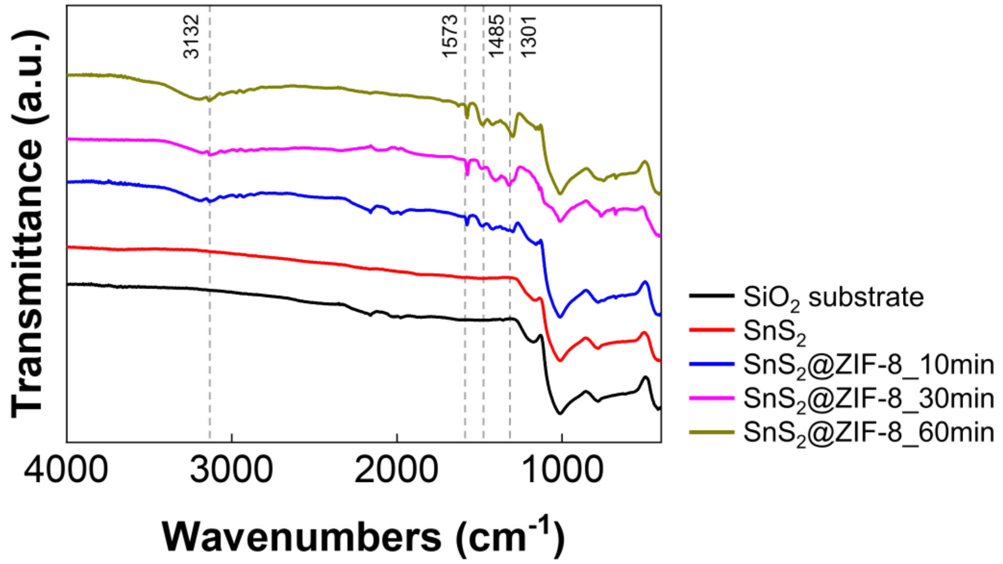


**Figure S2.** FT-IR spectra of $SiO_2$ substrate, pristine $SnS_2$, $SnS_2$@ZIF-8 10min, $SnS_2$@ZIF-8 30min, and $SnS_2$@ZIF-8 60min.

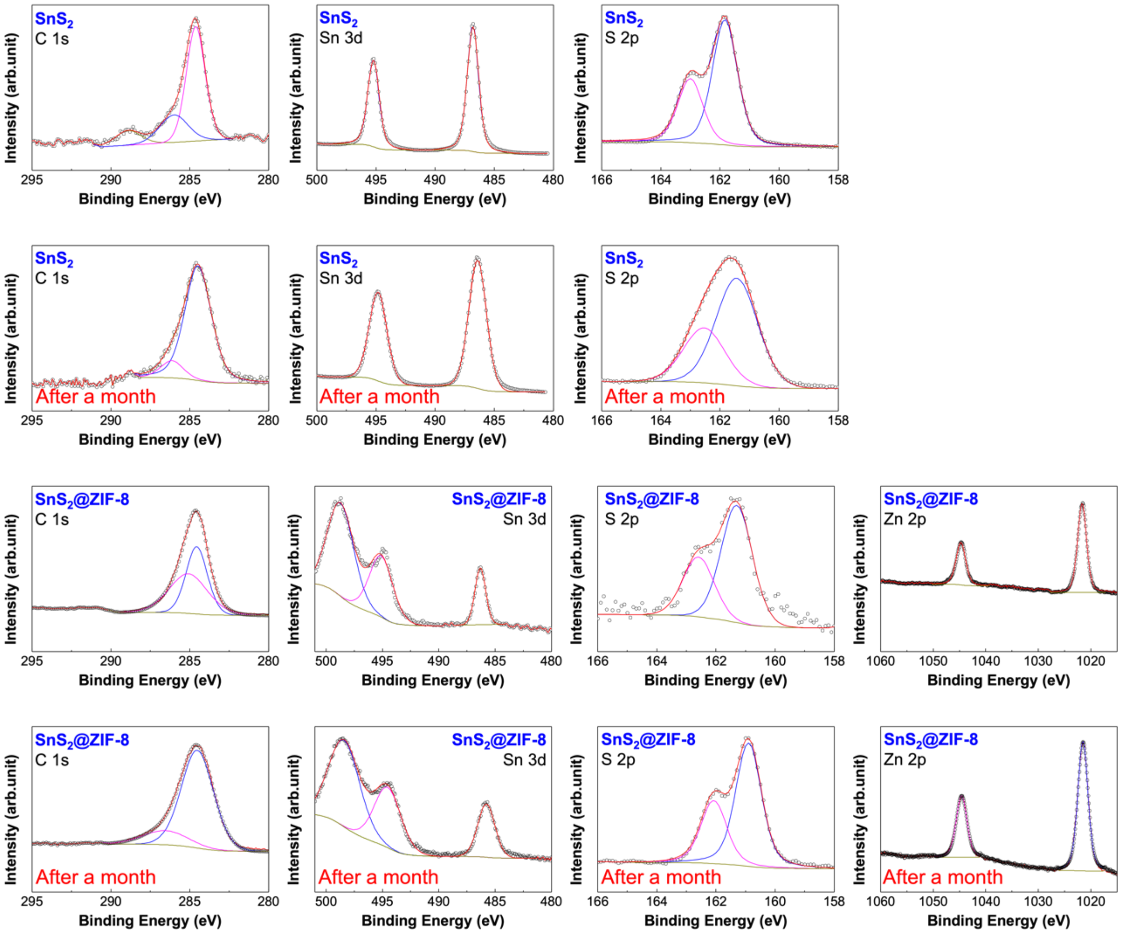


**Figure S3.** XPS analysis for the pristine $SnS_2$ and the $SnS_2$@ZIF-8 of the initial state and after a month of air exposure. XPS spectra of (1st row) the initial pristine $SnS_2$, (2nd row) the pristine $SnS_2$ after a month of contamination, (3rd row) the initial $SnS_2$@ZIF-8, and (4th row) the $SnS_2$@ZIF-8 after a month of contamination. Each column denotes specific atoms, (1st column) C, (2nd column) Sn, (3rd column) S, and (4th column) Zn. XPS spectra for Zn atom were only provided for the $SnS_2$@ZIF-8 sample due to the ZIF-8 layer.

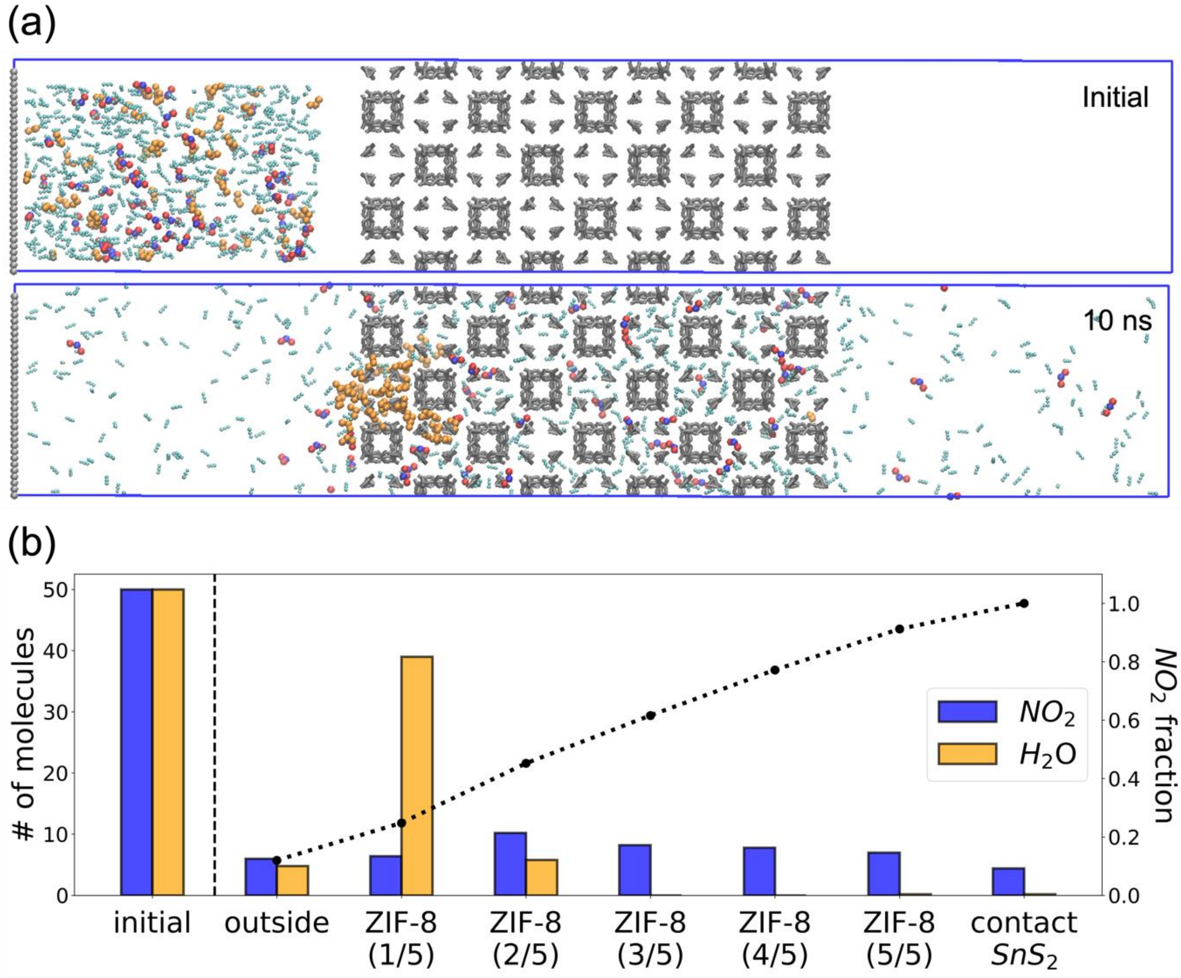


**Figure S4.** Additional MD simulations to calculate z-MSD of $NO_2$ and $H_2O$ molecules. (a) 500 $O_2$ (cyan), 50 $NO_2$ (blue/red) and 50 $H_2O$ (orange) molecules were initially packed at the left part of the ZIF-8 layer (top) and the snapshot after 10 ns was prepared (bottom). (b) The number of $NO_2$ and $H_2O$ molecules that exists at the specific region of the system (outside, ZIF-8 layer, and the region beyond ZIF-8 layer). Black line denotes cumulative $NO_2$ fraction.

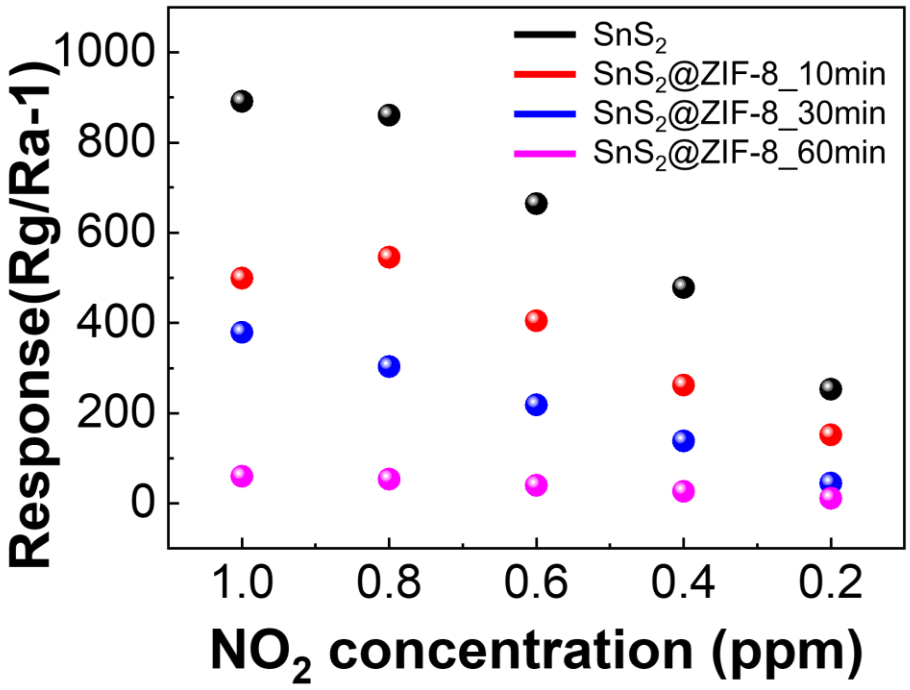


**Figure S5**. Calculated response for the samples (pristine $SnS_2$ $SnS_2$@ZIF-8_10min, $SnS_2$@ZIF-8_30min, and $SnS_2$@ZIF-8_60min) in respect to the concentration of $NO_2$ from 1 to 0.2 ppm.

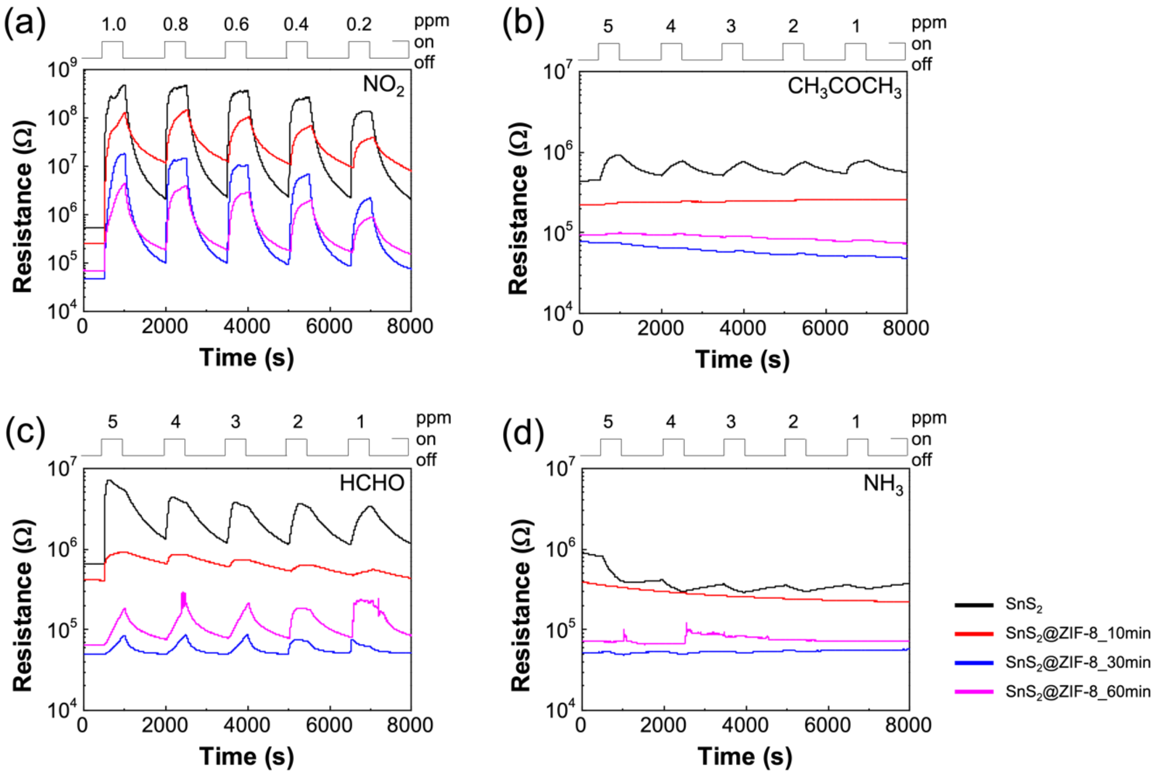


**Figure S6**. Dynamic response of the pristine $SnS_2$ and $SnS_2$@ZIF-8 with various growth time toward 4 different gas molecules ($NO_2$, $CH_3COCH_3$, HCHO, and $NH_3$). As written in manuscript, the concentration of (a) $NO_2$ was controlled from 1 to 0.2 ppm as the interval of 0.2 ppm. The concentration of (b) $CH_3COCH_3$, (c) HCHO, and (d) $NH_3$ were controlled from 5 to 1 ppm as the interval of 1 ppm. Black color denotes the pristine $SnS_2$, red color is the $SnS_2$@ZIF-8_10min, blue color is the $SnS_2$@ZIF-8_30min, and magenta color is the $SnS_2$@ZIF-8_60min.

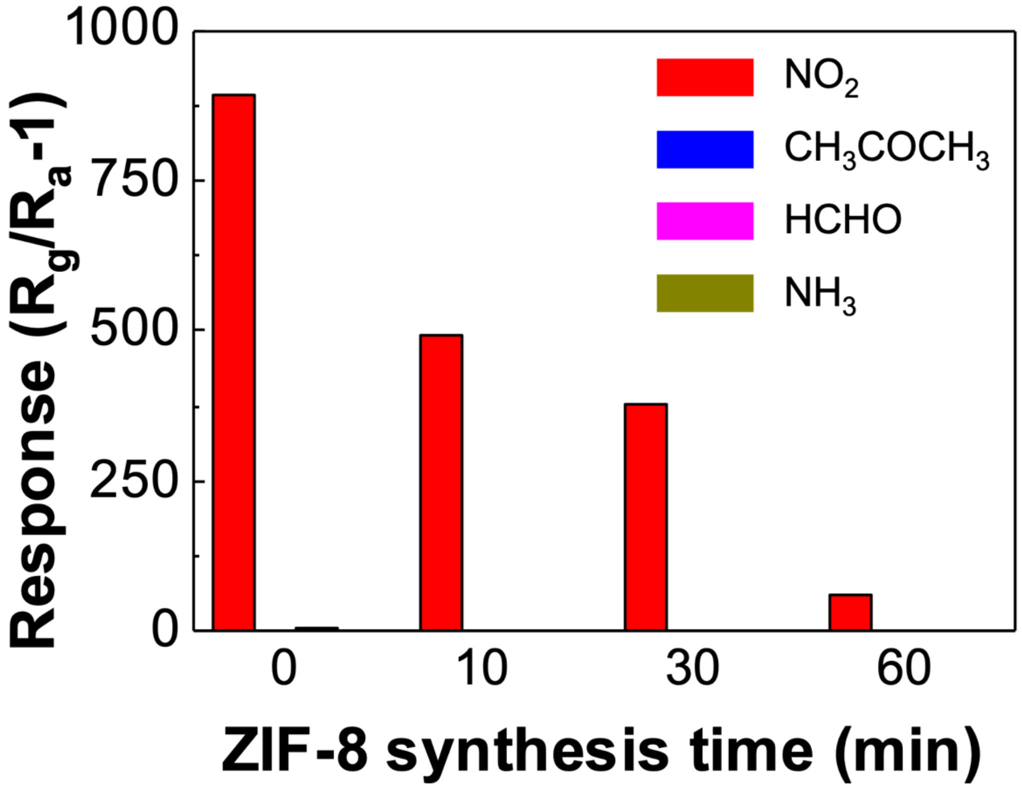


**Figure S7**. Changes in response according to the growth time difference for the ZIF-8 layer. Red color is $NO_2$, blue is $CH_3COCH_3$, magenta is HCHO, and khaki is $NH_3$. For all cases, all the other gas molecules ($CH_3COCH_3$, HCHO, $NH_3$) except $NO_2$ were not showed appreciable response.

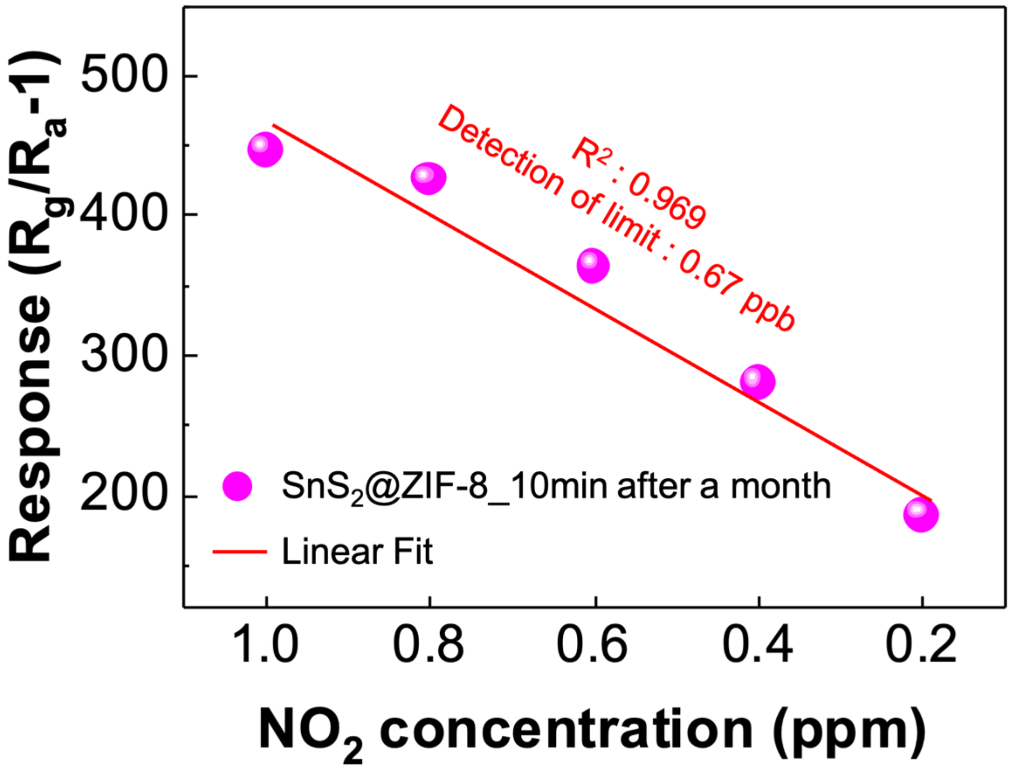


**Figure S8**. Response versus $NO_2$ concentration for the $SnS_2$@ZIF-8_10min sample after a month of air exposure. Linear fitting was conducted to find the detection of limit (DoL) and the $R^2$ score for fitted line is 0.969.

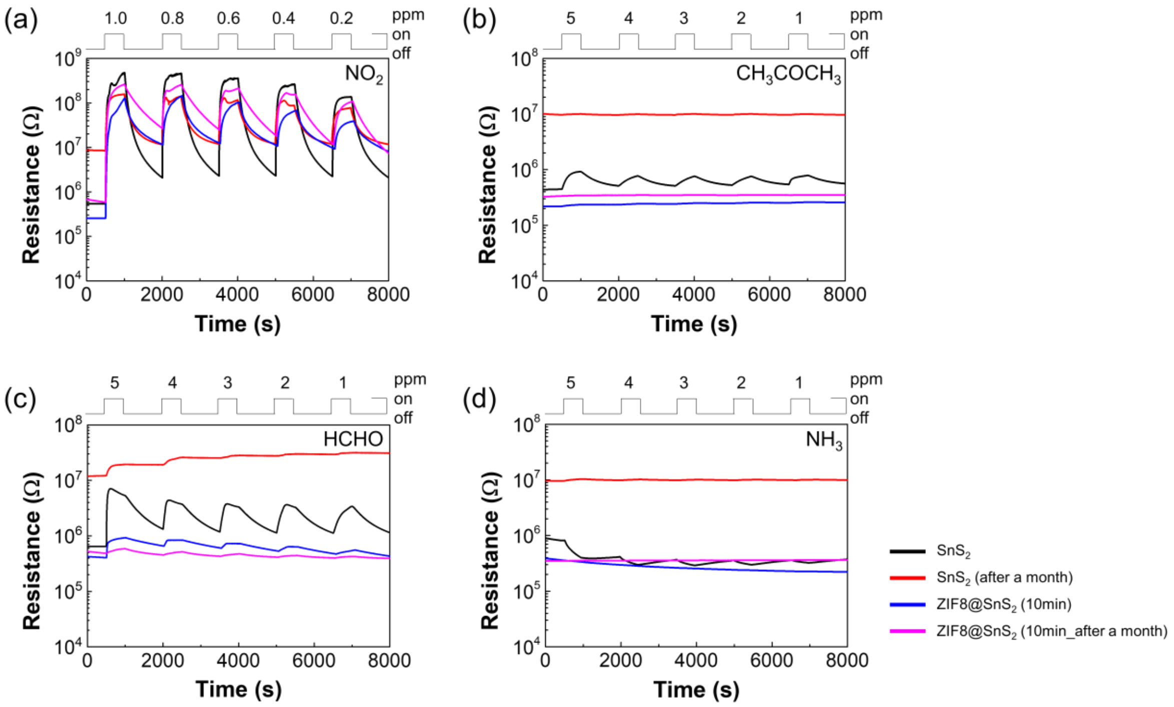


**Figure S9**. Dynamic response of the initial state of $SnS_2$ and $SnS_2$@ZIF-8_10min samples compared to the air exposed samples toward 4 different gas molecules ($NO_2$, $CH_3COCH_3$, HCHO, and $NH_3$). (a) The concentration of $NO_2$ was controlled from 1 to 0.2 ppm as the interval of 0.2 ppm. The concentration of (b) $CH_3COCH_3$, (c) HCHO, and (d) $NH_3$ were controlled from 5 to 1 ppm as the interval of 1 ppm.

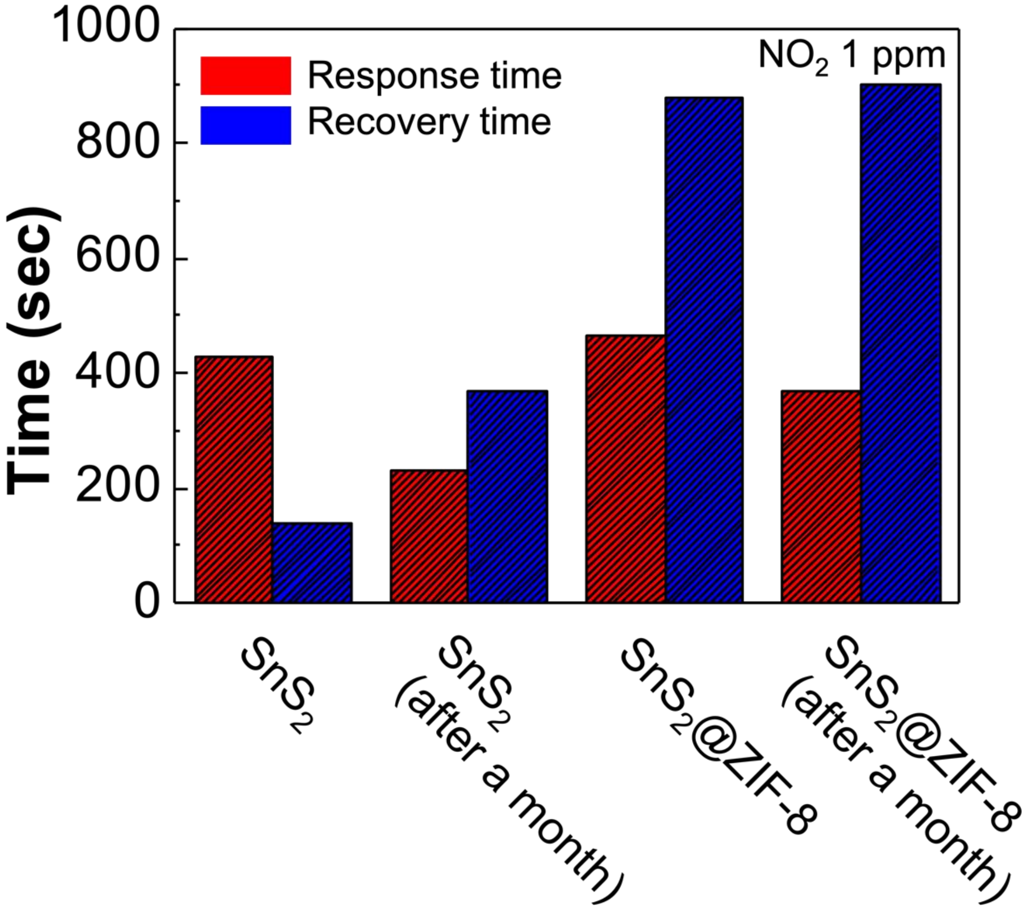


**Figure S10**. Response and recovery time for the samples (the pristine $SnS_2$ and the $SnS_2$@ZIF-8) with and without air exposure. Red color is response time and blue color is recovery time. The data was measured with $NO_2$ concentration as 1 ppm.

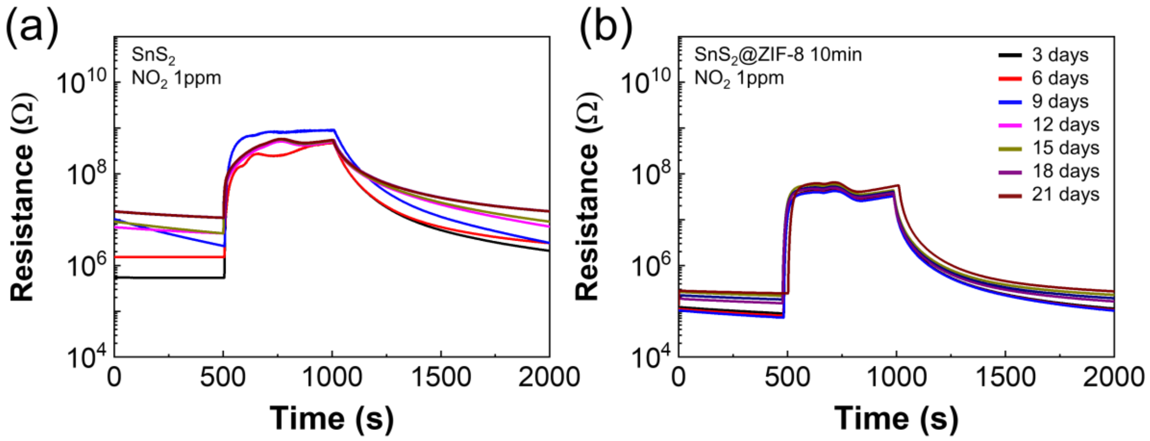


**Figure S11.** Time dependent $NO_2$ sensing response test for (a) pure $SnS_2$ and (b)$SnS_2$@ZIF-8_10 min

| Sensing element | $NO_2$(ppm) | Response ($R_g$/Ra) | Ref |
|---|---|---|---|
| ZnO | 4 | 3 | [1] |
| ZnO/g-$C_3N_4$ | 5 | 32 | [2] |
| $In_2O_3$/ZnO | 1 | 6 | [3] |
| $MoS_2$/ZnO | 0.1 | 12.8 | [4] |
| $SnO_2$ NW | 5 | 2.79 | [5] |
| $SnO_2$/MXene | 0.03 | 2.31 | [6] |
| $In_2O_3$ NW | 5 | 7.4 | [7] |
| $SnO_2$ | 10 | 10 | [8] |
| $MoS_2$ nanoflowers | 10 | 0.674 | [9] |
| $MoS_2$ | 2 | 0.01 | [10] |
| $MoS_2$/$SnO_2$ | 10 | 28% | [11] |
| $NbS_2$ nanosheet | 5 | 0.18 | [12] |
| BP | 1 | 6 | [13] |
| $MnPS_3$ | 1 | 4 | [14] |
| GO | 1 | 2.5 | [15] |
| $WS_2$/ZnS | 5 | 32 | [16] |
| $MoS_2$/ZnO | 5 | 30 | [17] |
| | 0.2 | 185 | |
| $SnS_2$@ZIF-8 | 0.6 | 363 | Our study |
| | 1 | 446 | |

**Table S1.** Comparison of previously reported $NO_2$ gas sensor.

**References**


[1] N. T. Thang, L. T. Hong, N. H. Thoan, C. M. Hung, N. Van Duy, N. Van Hieu, N. D. Hoa, *RSC Adv.* **2020**, *10*, 12759.
[2] K. S. Novoselov, A. K. Geim, S. V. Morozov, D. Jiang, M. I. Katsnelson, I. V. Grigorieva, S. V. Dubonos, A. A. Firsov, *Nature* **2005**, *438*, 197.
[3] J. K. Ellis, M. J. Lucero, G. E. Scuseria, *Appl. Phys. Lett.* **2011**, *99*, 261908.
[4] Y. Kim, K. C. Kwon, S. Kang, C Kim, T. H. Kim, S. -P. Hong, S. Y. Park, J. M. Suh, M. -J. Choi, S. Han, H. W. Jang, *Acs. Sensors.* **2019**, 4, 2395
[5] S. Cui, H. Pu, S. A. Wells, Z. Wen, S. Mao, J. Chang, M. C. Hersam, J. Chen, *Nat Commun* **2015**, *6*, 8632.
[6] R. Kumar, R. N. Jenjeti, S. Sampath, *ACS Sens.* **2020**, *5*, 404.
[7] M. Donarelli, S. Prezioso, F. Perrozzi, L. Giancaterini, C. Cantalini, E. Treossi, V. Palermo, S. Santucci, L. Ottaviano, *2D Mater.* **2015**, *2*, 035018.
[8] Y. Han, Y. Liu, C. Su, S. Wang, H. Li, M. Zeng, N. Hu, Y. Su, Z. Zhou, H. Wei, Z. Yang, *Sensors and Actuators B: Chemical* **2019**, *296*, 126666.
[9] Y. Han, D. Huang, Y. Ma, G. He, J. Hu, J. Zhang, N. Hu, Y. Su, Z. Zhou, Y. Zhang, Z. Yang, *ACS Appl. Mater. Interfaces* **2018**, *10*, 22640.
[10] R. Chen, J. Wang, L. Xiang, *Sensors and Actuators B: Chemical* **2018**, *270*, 207.
[11] H. Wang, J. Bai, M. Dai, K. Liu, Y. Liu, L. Zhou, F. Liu, F. Liu, Y. Gao, X. Yan, L. Geyu, *Sensors and Actuators B: Chemical* **2020**, *304*, 127287.
[12] R. K. Dharman, B. M. Francis, J. S. Ponraj, S. Muthuvijayan, R. K. Manavalan, S. Harisingh, S. Balasubramanian, S. C. Dhanabalan, *Journal of Materials Research and Technology* **2022**, *17*, 1760.
[13] A. R. Tripathy, A. Choudhury, A. Dash, P. Panigrahi, S. S. Kumar, P. P. Pancham, S. K. Sahu, S. Mallik, *Applied Surface Science Advances* **2021**, *3*, 100062.
[14] S. Park, S. An, Y. Mun, C. Lee, *ACS Appl. Mater. Interfaces* **2013**, *5*, 4285.
[15] S. Gasso, M. K. Sohal, A. Mahajan, *Sensors and Actuators B: Chemical* **2022**, *357*, 131427.
[16] C.-W. Chen, Y.-T. Chen, W. J. Tseng, *Materials Science and Engineering: B* **2021**, *265*, 115011.
[17] Y. Zhong, W. Li, X. Zhao, X. Jiang, S. Lin, Z. Zhen, W. Chen, D. Xie, H. Zhu, *ACS Appl. Mater. Interfaces* **2019**, *11*, 13441.
